# Supplementary figures and images for: Slow nucleosome dynamics set the transcriptional speed limit and induce RNA polymerase II traffic jams and bursts
Source: PLoS Comput Biol. 2022 Feb 10;18(2):e1009811. doi: 10.1371/journal.pcbi.1009811 (PMC8865691; doi:10.1371/journal.pcbi.1009811)

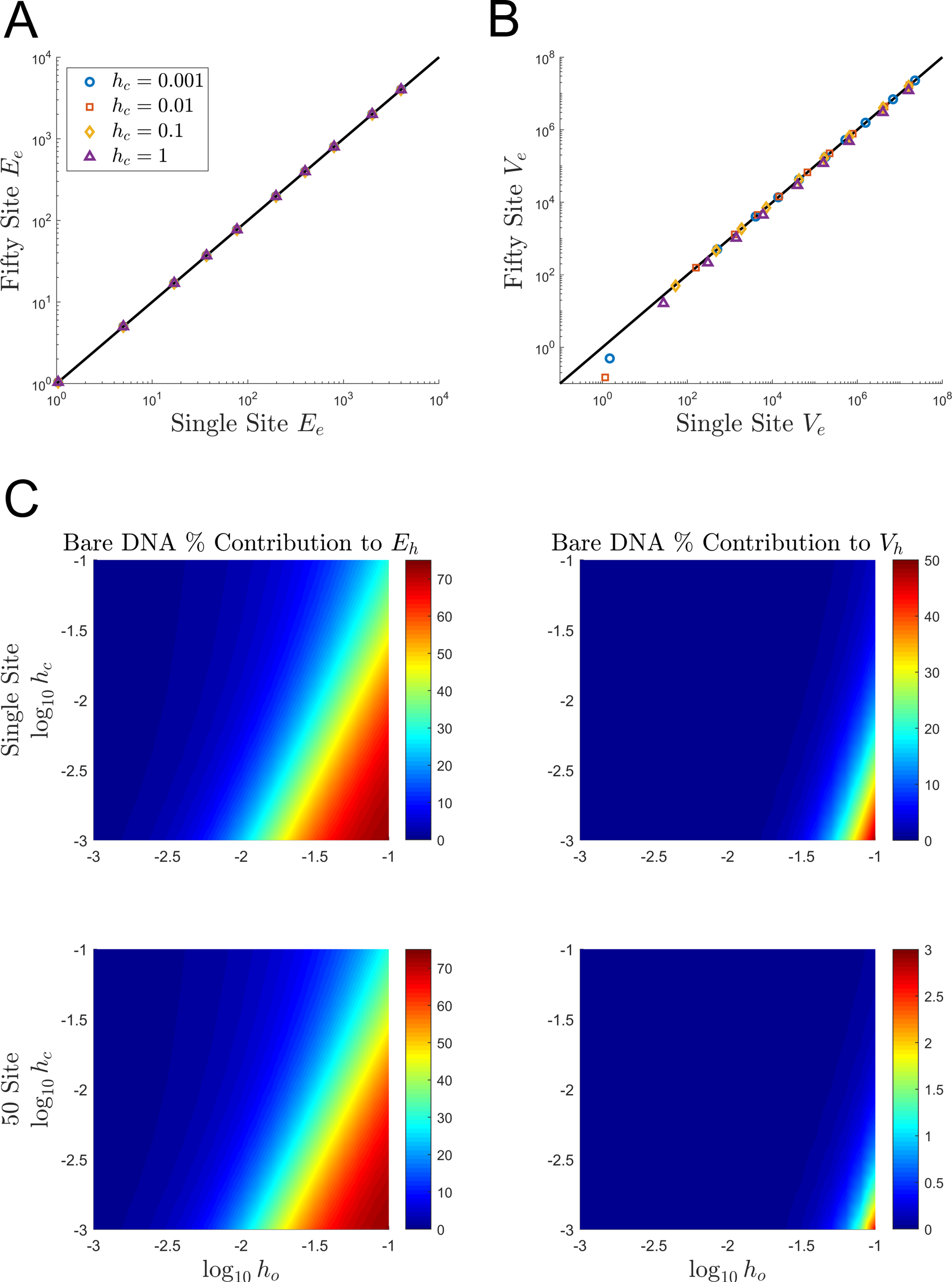

Supplement: S1 Fig — (A) compares the analytical results for the expected waiting time to enter a nucleosome between the single site and fifty site model showing that the results are mathematically identical. (B) compares the analytical results for the variance of the waiting time to enter a nucleosome between the single site and 50 site models confirming that they converge to each other in the limit as hc, ho→0. (A) and (B) are log-log plots with hc set to {0.001, 0.01, 0.1, 1} corresponding to red, blue, yellow, and purple markers respectively with ho adjusted to achieve the desired values of Ee and Ve. (C) Pseudo-color plots of the percent contribution of the bare DNA passage time to the mean and variance of first passage time to clear a nucleosome unit Eh (left) and Vh (right) for the single site (top) and 50 site (bottom) models. (TIF) [file pcbi.1009811.s001.tif]
