# Supplementary material for: Slow nucleosome dynamics set the transcriptional speed limit and induce RNA polymerase II traffic jams and bursts
Source: PLoS Comput Biol. 2022 Feb 10;18(2):e1009811. doi: 10.1371/journal.pcbi.1009811 (PMC8865691; doi:10.1371/journal.pcbi.1009811)
Supplement: Attachment — Submitted filename: ResponseToReviewers.docx [file pcbi.1009811.s002.docx]

We would first like to thank the reviewers for their insightful feedback into our manuscript and model. The questions have helped test the validity of our assumptions and have opened up interesting, new lines of inquiry for future research. We are therefore excited to present our new findings to you.

**Reviewer #1: Major Comments**

The article explains an interesting phenomenon of appearance of traffic jams on molecular highways as a result of slowing down of nucleosome transcriptional rates, which is an undesirable situation. The model used is ddTASEP which is one of the recent agent based models for studying gene transcription process. Overall the work is nice. I recommend the article for publication in this journal.

We would like to thank Reviewer #1 for the strong vote of confidence towards our work.

**Reviewer #2 Major Comments:**

1. The authors map the particle occupancy to 1 site, by scaling the transcription bubble to the unit length of the lattice in the TASEP. In reality, RNA polymerases move site by site with a rule of excluding the move if another polymerase is located L nt further, where L is the size of the bubble. These dynamics obey that of a TASEP with particles of extended size that has been studied in the context of translation and transcription (see Zia et al. for a review [https://doi.org/10.1007/s10955-011-0183-1](https://urldefense.com/v3/__https:/doi.org/10.1007/s10955-011-0183-1__;!!OToaGQ!4YLJ0t71LZEdUkQMheUcJUPqBWvWjSvEyrJNL8v2cpk_GNYhrEJOKhtLY6m9UUk$) and many other papers, included those cited below). It is also to be noted that at least two recent papers which I am aware of on this topic, e.g. Zuo and Chou arXiv:2103.05151 , and Turowski et al. [https://doi.org/10.1016/j.molcel.2020.06.002](https://urldefense.com/v3/__https:/doi.org/10.1016/j.molcel.2020.06.002__;!!OToaGQ!4YLJ0t71LZEdUkQMheUcJUPqBWvWjSvEyrJNL8v2cpk_GNYhrEJOKhtLzNJfUao$), use a TASEP model with extended particle size to model transcription. The approximation that consists of scaling a sum of moves with exponential rates to a single exponential jump is a priori wrong (a sum of independent exponential r.v.s is not an exponential r.v.), so the authors need to clarify why this simplification should be still relevant.

Reviewer #2 raised a rather insightful concern about the choice we made to represent polymerases as one-site particles on the ddTASEP lattice with each site corresponding to 50 base-pairs of DNA referencing Zia et al.’s review article on the extended body TASEP utilized to model protein translation  (<https://doi.org/10.1007/s10955-011-0183-1>). Most notably, Reviewer #2 pointed out the valid criticism that our proposed rescaling of 50 base-pairs to 1 site would lead to an over-estimation of the variance of the waiting time for polymerase advancement events.

This concern is completely justified. The waiting time for the sum of 50 single base pair steps at 50 bp/s would actually be governed by an $Erlang(k=50, \lambda=50)$ distribution in contrast to our naïve $Exponential(\lambda=1)$ distribution. Therefore, the first distribution would predict a variance of 0.02$s^{2}$ per site while our naïve distribution predicts $1s^{2}$ per site. Likely, the true distribution is an Erlang or Gamma distribution that exists somewhere between these two extremes. Regardless, the higher variance of our proposed exponential distribution will lead to more polymerase-polymerase collisions.

Instead of trying to consider simultaneously increasing the resolution of the lattice while also increasing the length of the polymerases, we decided to review the literature for results on single site, non-Markovian TASEPs. Referring to the work of Khoromskaia et al. (<https://arxiv.org/abs/1408.2139>) on non-Markovian exclusion processes, utilizing a more realistic waiting time distribution will reduce the variance in the hop rate leading to fewer polymerase-polymerase collisions. This, in turn, increases the bulk elongation rate and the flux since each polymerase’s velocity begins to approach the nominal value set by $q$. As Reviewer #2 expected, this phenomenon would have a significant impact if we were considering bare DNA.

However, we hypothesize that the dynamics of the nucleosomes will dominate both the mean and variance of the first passage time. Under the influence of the nucleosomes, we believe that the reduced variance in the hop time distribution of the polymerases (which would minimize polymerase-polymerase collisions) will be negligible relative to the variance added to the system by the nucleosome-induced pausing. To evaluate this hypothesis, we employed a two-pronged approach.

First, the transient state matrix from our initial Markov chain analysis was generalized to include 50 intermediate states to simulate single base pair resolution. Our calculations confirmed that mean first passage time for nucleosome entry was not altered regardless of the choice of resolution and that the variance of first passage time for the multi-step model converged to that of the single-step model in the limit as $h_{c}, h_{o}\to0$. This demonstrated that our choice of scale did not affect lead polymerase dynamics.

Second, we investigated the percentage of the mean first passage time and variance of first passage time of a nucleosome unit that was contributed by the bare DNA passage time versus the nucleosome entry step. For biologically interesting conditions when $h_{o}<h_{c}$, the bare DNA passage time contributes roughly 10% of the mean first passage time to clear a nucleosome in the single site model. With respect to the variance, the bare DNA passage time becomes even less significant. In the exponentially distributed waiting time/single site case, the bare DNA passage time contributes less than 10% of the variance to the overall passage time (excluding cases with $h_{o}\gg h_{c}$ which constitute effectively bare DNA).

Combining these two pieces of information, the key parameters that describe the pioneering polymerase first passage time statistics are robust with respect to changes of scale, and the reduction in variance due to a more accurate waiting time distribution leading to fewer polymerase-polymerase collisions is negligible relative to the new source of variance provided by the nucleosomes. Therefore, while a more accurate waiting time distribution would matter on bare DNA, we expect this effect to be negligible in the presence of nucleosomes.

The full analysis of this argument that has been sketched here has been added to the section deriving the first passage time statistics, and Supplemental Figure 1 was created to display these results.

The aforementioned papers of Zuo and Chou and Turowski et al. especially focus on some important aspects of RNA Pol dynamics, including backtracking and DNA torque. While these studies don’t require or assume a nucleosome free DNA in the modelling, I believe that the present paper is strongly limited in the biological interpretation, as there is no backtracking or heterogeneity in transcription (for example GC content biases the elongation rate), which are known to affect transcription dynamics. With the orders of magnitude involved, is it reasonable to consider a TASEP model that does not take these aspects into account?

This comment is extremely insightful, and we would like to thank the reviewer for helping us to open up an entirely new line of inquiry that we intend to pursue after the completion of this project.

First, nucleosomes are the strongest regulator of transcriptional dynamics. In cases where the DNA is completely depleted of histones (as occurs with loss of stem-loop-binding protein), cells show a global increase in transcriptional elongation rates, loss of almost all transcriptional pausing as if the polymerase were transcribing bare DNA, and altered patterns of co-transcriptional splicing (<https://www.pnas.org/content/112/48/14840>). Further, an emerging body of evidence is forming in the literature that implicates the role of enzymes such as BRD4 and DNMT1/DNMT3B as epigenetic drivers of cancer and epithelial to mesenchymal transition. BRD4 is a histone acetyltransferase associated with histone displacement, chromatin de-compactification, and cell cycle progression (<https://www.ncbi.nlm.nih.gov/pmc/articles/PMC4899182/>). BRD4’s upregulation has been associated with EMT in hepatocellular carcinoma and in salivary adenoid cystic carcinoma . DNMT’s are DNA methyltransferases that maintain gene silencing by hypermethylating CpG islands which encourages tighter nucleosome occupancy. Loss of DNMT activity leads to loss of nucleosomes on the promoter of tumor suppressor genes leading to their transcriptional activation (<https://www.ncbi.nlm.nih.gov/pmc/articles/PMC3898323/>).

Second, while static features such as elevated exon density, methylation, or elevated GC content are associated with pausing, these features induce pausing by modulating the wrapping dynamics of nucleosomes. Wang, Stein, and Ware demonstrated that nucleosomes have higher occupancy on regions of higher GC content and on exons (<https://arxiv.org/abs/1404.2487>). Jonkers, Kwak, and Lis demonstrated that increased exon density increased RNAPII pausing due to co-transcriptional splicing at intron-exon junctions (<https://elifesciences.org/articles/02407>). Collings, Waddel, and Anderson showed that methylated CpG sites (associated with transcriptional silencing) are more highly occupied by nucleosomes while unmethylated CpG islands are associated with polymerase recruitment and nucleosome depletion (<https://www.ncbi.nlm.nih.gov/pmc/articles/PMC5387343/>)

To address this heterogeneity, we have modified the ddTASEP’s core algorithm to allow for local variability in the polymerase advance rate $q_{i}$ and in the nucleosome wrapping dynamic rate constants $h_{c,m}$ and $h_{o,m}$. We have utilized our expanded model to simulate hypothetical genes that contain GC content variability, partially methylated CpG islands, and exons where pausing is known to occur. Subsequently, we utilized this algorithm to demonstrate that similar density patterns and throttling effects on the transcription flux could be achieved by modeling these features as either static defects or indirectly through their effects on nucleosomes. We demonstrated that through careful recalculation of $\gamma$ and rescaling of all the other parameters with respect to the slowest $q_{i}$ or fastest $h_{c,m}/$slowest $h_{o,m}$ that we could place upper and lower bounds on the throttled transcription flux based on the unperturbed flux prediction and the prediction assuming all sites had the properties of the slowest site.

This has been incorporated into the manuscript as the new Figure 9.

While the authors investigate the size and frequency of transcriptional bursts, I find surprising that they don’t relate their study to the widely used model of transcriptional burst induced by two state promoter (see review by Sanchez and Golding DOI: 10.1126/science.1242975 , or this more recent review doi: 10.1111/brv.12452 ), with many experimental papers working with this framework (including recent papers such as DOI: 10.1126/sciadv.aaz6699 or doi : 10.1038/s41467-021-24461-6) to explain burst size and frequency. While this mechanism controls the burst at initiation, does an elongation-controlled burst yields any difference, or could be better explaining some experimental observations?

The mechanism of transcriptional bursting resulting from promoter state switching has been investigated thoroughly before. In our study, we simply concentrate on the other mechanism that is associate with
nucleosome wrapping and unwrapping. As discussed in Introduction, nucleosomes are the primary regulators of transcriptional elongation. In cases where the DNA is completely depleted of histones (as occurs with loss of stem-loop-binding protein), cells show a global increase in transcriptional elongation rates, loss of almost all transcriptional pausing as if the polymerase were transcribing bare DNA, and altered patterns of co-transcriptional splicing.

An emerging body of evidence suggests that epithelial to mesenchymal transition (EMT) is regulated by BRD4, a histone acetyltransferase associated with histone displacement, chromatin de-compactification, and cell cycle progression. Histone displacements upregulate on a time scale of days with changes in expression of  EMT drivers SNAI1, TWIST1, and ZEB1 <https://journals.physiology.org/doi/full/10.1152/ajplung.00224.2016>. We thus think that promoter
state switching and nucleosome pausing are both important mechanisms regulating transcription elongation and flux.

We have also added a brief section associated with Figure 8 discussing how a two-state model could be interpreted through the framework of the TASEP.

4. In the section where the parameter gamma is introduced and studied, as the modified elongation rate due to histone dynamics, I find the derivation of the mean and variance for the FPT not very interesting (it is a quite basic calculation using continuous time Markov chain);

We agree with the reviewer that this section is not very interesting. However, after much deliberation, we opted to keep the derivation to avoid confusing the audience in the later passages. First, while the main purpose of this section is to derive the mean first passage rate $\gamma$, there are many other important properties defined within this section ($E_{e}, E_{h}, V_{e}, V_{h}, D_{e}, MFPT, VFPT)$ that the reader needs to familiarize themselves with to understand subsequent figures throughout the paper, especially the index of dispersion of the entry time $D_{e}$ which is directly involved in the burst modeling. Second, in our experience presenting this work at meetings, it is frequently unintuitive to the audience that $\gamma$ is not a free parameter but that it is directly calculated from the nucleosome rate constants $h_{o}$ and $h_{c}$. Thus, having this derivation explicitly shown could be beneficial to the reader since it directly reinforces the notion that the nucleosome dynamics are controlling the first passage rate. Finally, we had to extend our existing analysis to consider the possibility of spatially extended polymerases in order to justify the validity of the coarse-graining assumption posed in the first comment by Reviewer #2.

the relevance of this part is stated at the very end of the paper, but with no evidence or reference that would support the authors claim, so I think this should either be a supplement, or revised to make it more relevant with actual experiments and applications.

We have removed the discussion of hypothetical experiments that could be used to measure the nucleosome dynamic rate constants and replaced it with a more interesting discussion of new results derived from Reviewer #2’s line of inquiry into other gene features.

Technically, the authors provide some heuristics to infer the flux and densities that yield a partial phase diagram in gamma. While the results are sound, I also suspect they can also be quite naturally derived from a mean-field approximation to solve the master equation associated with the process (some formulas seem quite close to the TASEP with extended particles, with some correspondence between the parameters gamma and the particle size DOI:10.1088/0305-4470/36/8/302). This would confirm the authors result and help to interpret the limitations that seem to numerically appear in a certain range of the parameters.

We were familiar with the article by Lakatos and Chou that Reviewer #2 mentioned. Ideally, we would have liked to use a mean-field approach to solve the Chemical Master Equation for the core transcription observables $J, \rho,$ and $v$ and to locate the phase transition in parameter space. However, this approach has significant limitations in the unique context of our model.

Based on the model formulation in Equations 1 – 6, it is clear that the system’s chemical master equation would involve the products of up to four site occupancies. Therefore, when taking the ensemble average of the site occupancies, quadratic (e.g. $\langle s_{i}s_{i+1}\rangle)$ and cubic mixed moments (e.g. $\langle s_{i+1}s_{i+2}s_{i+3}\rangle)$ appear in the master equation. (Note that quartic moments such as $\langle h_{m}s_{i+1}s_{i+2}s_{i+3}\rangle$ vanish due to the exclusionary binding constraint.) Under the mean-field approximation (also referred to as the random phase approximation for the TASEP), these mixed moments can be factored as

$$\left\langle s_{i}\left( 1-s_{i+1} \right) \right\rangle\approx\left\langle s_{i} \right\rangle\left\langle1-s_{i+1} \right\rangle=\rho_{i}\left( 1-\rho_{i+1} \right)$$

which implies that each site of the classical TASEP comes to a steady state value and that no site occupancies are correlated with each other. This assumption clearly fails for the proposed ddTASEP with extended dynamic defects and exclusionary defect binding as shown in Figs 2E and 2F which depict the strong inter-site correlations. These correlations violate the assumption of the statistical independence of neighboring lattice sites used to eliminate the covariance term when calculating the expectation value of a product of non-independent random variables.

While cluster mean-field methods do exist for tracking the higher order moments of the TASEP, they are rarely implemented beyond second order in the literature. Further, even if we derived these equations, they would not be able to be solved analytically since they are a highly nonlinear system of ODEs and could only be integrated numerically. We believe that the numerical solutions will provide minimal insight into the source of the discrepancy between the simulated values of the transcription observables and the heuristic expressions we proposed. Therefore, we abandoned this approach.

These points have been included in the manuscript in the transition between the derivation of the first passage rate $\gamma$ and the presentation of the heuristic expressions for $J, \rho$, and $v$ on Pages 24 – 25.

**Reviewer #2 Minor Comments:**

There should be a rigorous mathematical definition of the ddTASEP model in the methods sections

We have added this to the first section of the results on pages 13 – 14.

Fig 4: In the IL region, typo on J

We removed the extra unclosed parentheses off of this figure, and we corrected a previously unnoticed issue with the figure axes labels.
